# The last frontier for global non-communicable disease action: The emergency department—A cross-sectional study from East Africa

**DOI:** 10.1371/journal.pone.0248709

**Published:** 2021-04-02

**Authors:** Christine Ngaruiya, Mbatha Wambua, Thomas Kedera Mutua, Daniel Owambo, Morgan Muchemi, Kipkoech Rop, Kaitlin R. Maciejewski, Rebecca Leff, Mugane Mutua, Benjamin Wachira

**Affiliations:** 1 Department of Emergency Medicine, Yale University, New Haven, CT, United States of America; 2 Department of Emergency Medicine, Muhimbili University of Health and Allied Sciences, Dar-es-Salaam, Tanzania; 3 The Nairobi West Hospital, Nairobi, Kenya; 4 The Aga Khan University Hospital, Nairobi, Kenya; 5 Kenyatta University Teaching, Referral & Research Hospital, Nairobi, Kenya; 6 University of Nairobi, Nairobi, Kenya; 7 Yale Center for Analytical Sciences, Yale School of Public Health, New Haven, CT, United States of America; 8 School of Medicine, Faculty of Health Sciences, Ben-Gurion University of the Negev, Beer -Sheva, Israel; 9 Elburgon, Kenya; 10 Accident and Emergency Department, The Aga Khan University Hospital, Nairobi, Kenya; Chiang Mai University Faculty of Medicine, THAILAND

## Abstract

**Introduction:**

Deaths due to non-communicable diseases (NCDs) have surpassed those due to communicable diseases globally and are projected to do so in Africa by 2030. Despite demonstrated effectiveness in high-income country (HIC) settings, the ED is a primary source of NCD care that has been under-prioritized in Africa. In this study, we assess the burden of leading NCDs and NCD risk factors in Kenyan Casualty Department patients to inform interventions targeting patients with NCDs in emergency care settings.

**Materials and methods:**

Using the WHO STEPwise approach to surveillance (STEPS) tool and the Personal Health Questionnaire (PHQ-9), we conducted a survey of 923 adults aged 18 and over at Kenyatta National Hospital Emergency Department (KNH ED) between May-October 2018. Age, income, household size(t-test), sex, education, marital status, work status, and poverty status (chi-squared test or fisher’s exact test) were assessed using descriptive statistics and analyzed using covariate-adjusted logistic analysis.

**Results:**

Over a third of respondents had hypertension (35.8%, n = 225/628), 18.3% had raised blood sugar or diabetes (18.3%, n = 61/333), and 11.7% reported having cardiovascular disease (11.7%, n = 90/769). Having lower levels of education was associated with tobacco use (OR 6.0, 95% CI 2.808–12.618, p < 0.0001), while those with higher levels of education reported increased alcohol use (OR 0.620 (95% CI 0.386–0.994, p = 0. 0472). While a predominant proportion of respondents had had some form of screening for either hypertension (80.3%, n = 630/772), blood sugar (42.6%, n = 334/767) or cholesterol (13.9%, n = 109/766), the proportion of those on treatment was low, with the highest proportion being half of those diagnosed with hypertension reporting taking medication (51.6%, n = 116/225).

**Conclusions:**

This study establishes the ED as a high-risk population with potential for high impact in East Africa, should targeted interventions be implemented. Comprehension of the unique epidemiology and characteristics of patients presenting to the ED is key to guide care in African populations.

## Introduction

Non-Communicable Diseases (NCDs) annually constitute more than 70% of deaths worldwide [1]. Furthermore, current disease trends suggest worsening of the situation over the next decade, with the WHO projecting 55 million deaths from NCDs annually by 2030 [2]. NCDs have surpassed communicable diseases as the lead cause of death in all continents except Africa, where NCD-related deaths are nevertheless projected to surpass deaths from communicable diseases, maternal and perinatal conditions, and nutritional deficiencies by 2030 [1]. Eighty-percent of deaths from NCDs occur in low- and middle- income countries (LMICs) with the majority of these occurring prematurely [2].

The 2013–2020 WHO global action plan for NCDs highlights such targets as: reduction in premature mortality secondary to cardiovascular disease, cancer, diabetes, and chronic respiratory disease; reduction in harmful use of alcohol; reduction in prevalence of tobacco use; reduction in prevalence of raised blood pressure, and increased prevalence of eligible people on appropriate therapy for cardiovascular disease prevention [2]. Interventions in high-income countries (HICs) have demonstrated the effectiveness of the Emergency Department (ED) in addressing all of these targets, including tobacco cessation, alcohol cessation, and use of navigators to improve compliance among diabetics, among others [3–5]. The ED is an optimal setting for these and other novel interventions targeting NCDs.

The ED is also the primary site for presentation of patients with acute NCD-related complications (such as acute coronary syndrome, strokes, diabetic ketoacidosis or asthma exacerbations), mental illness and injury-related complaints [6]. All of these conditions require timely and effective management in order to mitigate long-term effects of disease. The importance of studying NCDs in the ED and developing high yield interventions to improve management of those presenting with NCD-related acute illness to prevent downstream effects, is paramount.

In this study, we assess the burden of leading NCDs and NCD risk factors in Kenyan Casualty Department patients, in order to inform development of hospital and clinical policies, educational interventions for practitioners on management of NCDs in the emergency setting, and novel interventions targeting patients with NCDs in the Casualty setting. This is the largest epidemiological study on NCDs in an African Emergency Department that we are aware of.

## Materials and methods

### Study design

This was a prospective, cross-sectional study using surveys administered by data collectors to patients accessing care in the ED of the largest tertiary referral hospital in Kenya and East Africa, Kenyatta National Hospital. The WHO Stepwise Approach to Surveillance (STEPS) tool was used to assess burden of NCDs (particularly hypertension, diabetes and cardiovascular disease in this study), as well as prevalence of NCD risk factors (tobacco and alcohol in this study), and the Personal Health Questionnaire (PHQ-9) was used to assess prevalence of depression [7,8]. These diseases and risk factors were prioritized given their relative contribution to the global burden of NCD related morbidity and mortality [1], as well as because of established effectiveness to affect these particular conditions in the ED as demonstrated in HIC settings [3–5]. The tools are both internationally validated and publicly available. A Swahili version of the STEPS tool was obtained from the Kenya National STEPS study team [11]. A Swahili version of the PHQ-9 that has been validated in a Kenyan population by Omoro et al [8] was used for the latter. Results from the PHQ-9 tool are presented elsewhere.

Two data collectors were hired to assist with administration of surveys. Local data collectors were used who were familiar with the patient population and spoke the native languages. The survey was verbally administered to account for potential barriers with illiteracy, with responses indicated on electronic tablets. Surveys were loaded on to the Kobo software platform for ease of use. The surveys were offered in English and in the national language, Kiswahili. All patients provided written informed consent. This study received approval from the Institutional Review Board at Yale University (IRB Protocol ID 2000022697) and from the Kenyatta National Hospital/ University of Nairobi Ethics Review Committee (study registration No. A&E/034/201).

### Sample size and population

**Fig 1:**
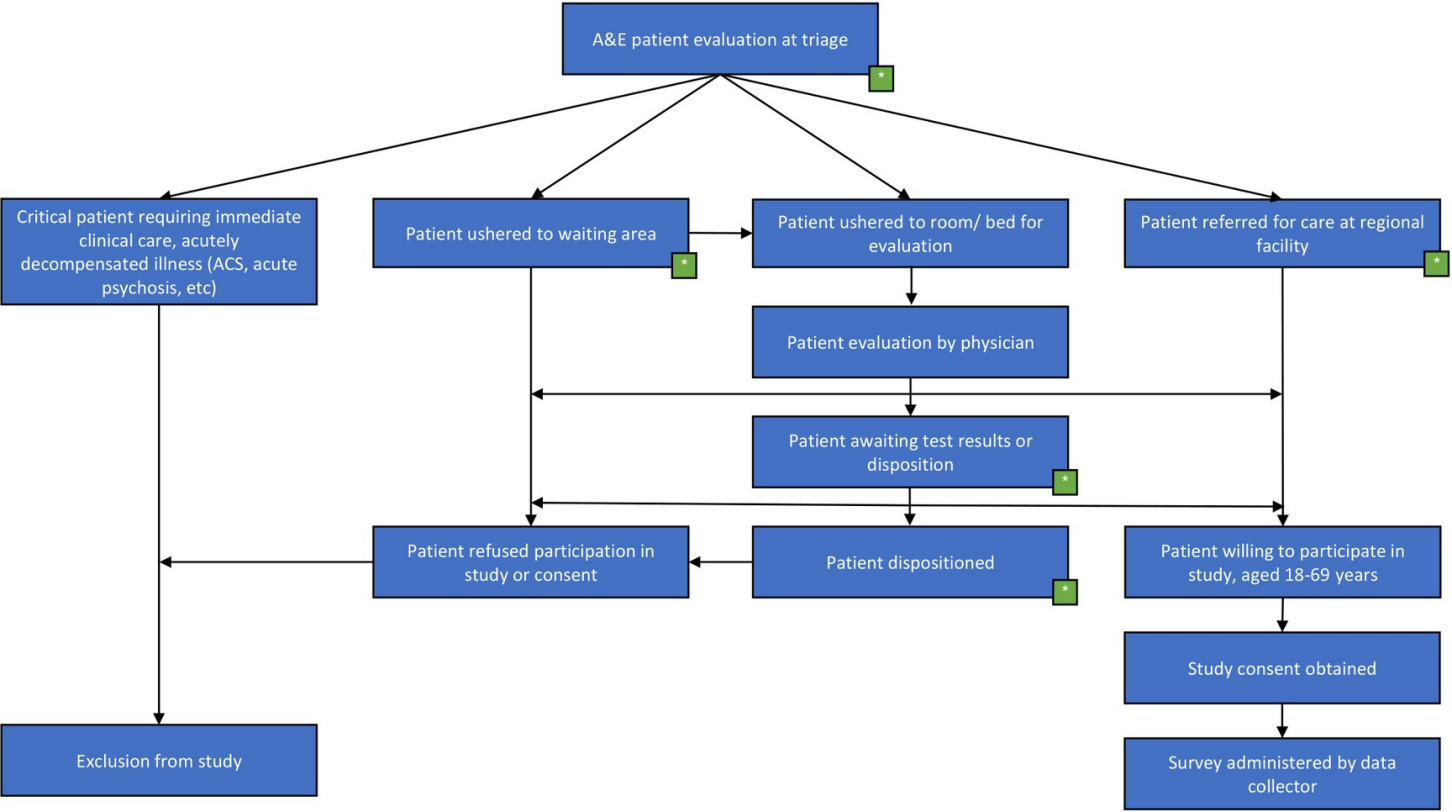
ED flow diagram/protocol. Key: A&E–Accident & Emergency Dept, ACS- Acute Coronary Syndrome, Green box–phase of care for approach by data collector.

Kenyatta National Hospital (KNH), located in Nairobi, is the lead referral hospital with a catchment of 3 million people across East Africa. It is the largest ED in East Africa seeing a wide variety of nationals including Kenyans and other East Africans, “medical tourists,” and refugees from across Africa. This was the study site. An estimate for the total number of patients seen at the KNH ED in a 3-month period (2014–2015) is between 14,956 and 23, 951, according to Myers et al [9]. No additional estimate is available to date from the facility or in the literature. Based on this, we had a target sample size of 2,400 or 10% of the upper estimate of total number of presentations over that time period (3 months), in line with standard pilot proportions [10]. We aimed to recruit these 2,400 participants similarly during a 3-month period (May 2018-July 2018). However, coinciding with the onset of data collection for our study, national referral protocols changed resulting in a substantial drop in the patient volume at the KNH ED. Therefore, in lieu of our original sampling approach, we instead extended data collection to the entirety of the funding period (6 months) and used a convenience sample of patients that were collected on randomized days, and across randomized time blocks for data collection (0800–1200, 1200–1600, and 1600–2000), to help ensure data collection across different days and time periods for presentation to the ED. Using this approach, we recruited patients presenting to the ED during the extent of the study period (May 2018-October 2018), and included all patients meeting our inclusion criteria. Patients were recruited in the ED for involvement in the study either after triage, during the waiting period prior to being evaluated by a clinician or during the period of time after when they had already been assessed and were awaiting disposition (Fig 1). This approach was used to help minimize interruptions to workflow in the Department. Patients who agreed to participate were then interviewed in a room adjacent to the ED clinical space. All patients aged 18 years old or older were considered, which mirrored the age group used in the 2015 Kenya STEPS study [11]. Patients who refused or were unable to provide informed consent were excluded.

### Measures

The analytical sample includes all those who consented to be surveyed. Not all participants answered all questions. Demographic information including age and sex (male, female) were collected. Marital status was categorized as married (currently married or cohabitating), formerly married (divorced, separated, widowed), never married. Participants were asked their highest education level, which was then categorized as secondary school or above (Secondary school completed, High school completed, College/University completed, Post graduate degree), primary school completed, less than primary school completed (less than primary school, no formal schooling). To collect employment information, participants were asked “which of the following best describes your main work status over the past 12 months?” Responses were then categorized as: Employed (Government employee, Non-government employee), Not employed (Homemaker, retired, non-paid, Unemployed (able to work), Unemployed (unable to work), Student, Self-employed.

To gain information about income, participants were asked “Taking the past year, can you tell me what the average earnings of the household have been?” They were also asked “How many people older than 18 years, including yourself, live in your household?” These variables were then used to create an indicator for household poverty, using the WHO standard [12]. The household was considered to be in poverty if household income/household size < 69397.5 KSH (given a poverty cut-off of $1.90 per person per day ($1.9 * 365.25days * 100KShs/$ = 69397.5 KShs). Medication use was determined by asking “are you currently taking [medication]” for a given health disorder. The denominator was restricted only to those who were told they had the related condition by a doctor or other health worker.

### Statistical analysis

First, descriptive statistics were generated to characterize the overall population. Mean, standard deviation, median, range are presented for numerical variables, percentages are presented for categorical variables, and number missing are presented for all. Bivariate analyses were next conducted for each outcome of interest. Variables were compared by age, income, household size (t-test), sex, education, marital status, work status, and poverty status (chi-squared test or fisher’s exact test). Finally, multivariate analyses were conducted using covariate-adjusted logistic analysis. P < 0.05 was considered statistically significant. A table is included with all statistically significant relationships and our outcomes of interest (Table 15). Analysis was conducted using SAS 9.4 (SAS Institute, Inc., Cary, NC).

## Results

There were a total of 923 total respondents, of which 784 (84.9%) provided consent. The mean age was 35y (+/- 13.0y), with a range from 18y to 88y. The majority of respondents were male (61.2%, n = 480/784) (see Table 1). More than half of respondents had completed high school (secondary school) or higher (59.4%, n = 466/784). The predominance of respondents reported being married (54.6%, n = 428/784). Majority reported being self-employed (41.2%, n = 323/784), and 19.9% reported being unemployed (n = 156/784). The average reported annual household income was Kenya Shillings (Kshs) 237,888.6, or approximately 2,379 USD, with 30.6% falling below the World Bank international poverty line of 1.90 USD per day (n = 240/784).

**Table 1:** Summary table on participants included in the study.

	Total (N = 784)
**Age**	
Missing (not Missing)	17 (767)
Mean (SD)	35.23 (12.95)
**Household income**	
Missing (not Missing)	268 (516)
Mean (SD)	237888.61 (268912.02)
**Household size**	
Missing (not Missing)	9 (775)
Mean (SD)	2.65 (1.91)
**Sex**	
Missing	2 (0.26%)
Female	302 (38.52%)
Male	480 (61.22%)
**Education Level**	
Missing	5 (0.64%)
Less than primary school or none	102 (13.01%)
Primary school completed	211 (26.91%)
Secondary school or above	466 (59.44%)
**Marital Status**	
Missing	7 (0.89%)
Formerly Married	68 (8.67%)
Married	428 (54.59%)
Never married	281 (35.84%)
**Work Status**	
Missing	8 (1.02%)
Employed	202 (25.77%)
Self-employed	323 (41.20%)
Student	95 (12.12%)
Unemployed	156 (19.90%)
**Poverty**	
Missing	269 (34.31%)
N	275 (35.08%)
Y	240 (30.61%)
**Have you ever been told by a doctor or other health worker that you have raised blood pressure or hypertension?**	
Missing	156 (19.90%)
No	403 (51.40%)
Yes	225 (28.70%)
**Have you ever been told by a doctor or other health worker that you have raised blood sugar or diabetes?**	
Missing	451 (57.53%)
No	272 (34.69%)
Yes	61 (7.78%)
**Have you ever had a heart attack or chest pain from heart disease (angina) or a stroke (cerebrovascular accident or incident)?**	
Missing	15 (1.91%)
No	679 (86.61%)
Yes	90 (11.48%)
**Have you ever been told by a doctor or other health worker that you have raised cholesterol?**	
Missing	675 (86.10%)
No	79 (10.08%)
Yes	30 (3.83%)
**Ever used tobacco**	
Missing	10 (1.28%)
No	476 (60.71%)
Yes	298 (38.01%)
**Current tobacco user**	
Missing	130 (16.58%)
No	535 (68.24%)
Yes	119 (15.18%)
**Have you ever consumed any alcohol such as beer, wine, or other spirits?**	
Missing	10 (1.28%)
No	297 (37.88%)
Yes	477 (60.84%)
**Have you consumed any alcohol within the past 12 months?**	
Missing	308 (39.29%)
No	200 (25.51%)
Yes	276 (35.20%)
**During the past 30 days, did someone smoke in your home?**	
Missing	10 (1.28%)
No	458 (58.42%)
Yes	316 (40.31%)
**During the past 30 days, did someone smoke in closed areas in your workplace (in the building, in a work area or a specific office)?**	
Missing	8 (1.02%)
Don’t work in a closed area	109 (13.90%)
No	293 (37.37%)
Yes	374 (47.70%)
**Have you ever had your blood pressure measured by a doctor or other health worker?**	
Missing	12 (1.53%)
No	142 (18.11%)
Yes	630 (80.36%)
**Have you ever had your blood sugar measured by a doctor or other health worker?**	
Missing	17 (2.17%)
No	433 (55.23%)
Yes	334 (42.60%)
**Have you ever had your cholesterol (fat levels in your blood) measured by a doctor or other health worker?**	
Missing	18 (2.30%)
No	657 (83.80%)
Yes	109 (13.90%)
**In the past two weeks, have you taken any drugs (medication) for raised blood pressure prescribed by a doctor or other health worker?**	
Missing	559 (71.30%)
No	109 (13.90%)
Yes	116 (14.80%)
**Are you currently taking insulin for diabetes prescribed by a doctor or other health worker?**	
Missing	723 (92.22%)
No	36 (4.59%)
Yes	25 (3.19%)
**In the past two weeks, have you taken any oral treatment (medication) for raised total cholesterol prescribed by a doctor or other health worker?**	
Missing	754 (96.17%)
No	19 (2.42%)
Yes	11 (1.40%)
**Are you currently taking aspirin regularly to prevent or treat heart disease?**	
Missing	694 (88.52%)
No	69 (8.80%)
Yes	21 (2.68%)
**Are you currently taking statins (Lovastatin/Simvastatin/Atorvastatin or any other statin) regularly to prevent or treat heart disease?**	
Missing	698 (89.03%)
No	71 (9.06%)
Yes	15 (1.91%)
	Total (N = 784)
**During the past 12 months, have you tried to stop smoking?**	
Missing	680 (86.73%)
No	45 (5.74%)
Yes	59 (7.53%)
**Have you stopped drinking due to health reasons, such as a negative impact on your health or on the advice of your doctor or other health worker?**	
Missing	584 (74.49%)
No	135 (17.22%)
Yes	65 (8.29%)

More than a third of respondents reported being told they had elevated blood pressure or hypertension by a health worker (35.8%, n = 225/628) (see Table 2). The average age of those diagnosed was 41.7y (+/- 14.5y), with a female predominance (56.25%, n = 126/225). The average reported income among those diagnosed was 2,406.85 USD, and most had completed high school (53.33%, n = 120/225) (see Tables 1 and 2). In the multivariate analysis, only age, sex, and being below the poverty line were predictors of likelihood of having been diagnosed with hypertension (Tables 3 and 15). For every 1-year increase in age, the odds of having been told by a doctor or other health worker that they had hypertension increased by 0.066 (6.6%) (95% CI 1.041–1.092, p <0.0001). Women had more than double the odds of being told they had hypertension as compared to men (OR 2.335, 95% CI 1.470–3.707, p = 0.0003). Those who were below the poverty line had 0.607 (95% CI 0.372–0.992, p = 0.0462) times the odds of having been told by a doctor or other health worker that they had hypertension, compared to those who were not.

**Table 2:** Sociodemographic characteristics of respondents told by a doctor or other health worker that they had raised blood pressure or hypertension.

	No (N = 403)	Yes (N = 225)	Total (N = 628)	P Value
**Age**				
Mean (SD)	33.57 (11.82)	41.69 (14.48)	36.51 (13.41)	<0.001***
**Household income (Ksh)**				
Mean (SD)	255320.72 (298334.54)	240685.90 (276783.29)	249711.30 (290005.41)	0.62
**Household size**				
Mean (SD)	2.60 (2.03)	2.96 (2.04)	2.73 (2.04)	0.034*
**Sex**				
Female	149 (37.06%)	126 (56.25%)	275 (43.93%)	<0.001***
Male	253 (62.94%)	98 (43.75%)	351 (56.07%)	
**Education Level**				
Less than primary school or none	34 (8.48%)	45 (20.00%)	79 (12.62%)	<0.001***
Primary school completed	107 (26.68%)	60 (26.67%)	167 (26.68%)	
Secondary school or above	260 (64.84%)	120 (53.33%)	380 (60.70%)	
**Marital Status**				
Formerly Married	24 (6.02%)	31 (13.78%)	55 (8.81%)	<0.001***
Married	219 (54.89%)	140 (62.22%)	359 (57.53%)	
Never married	156 (39.10%)	54 (24.00%)	210 (33.65%)	
**Work Status**				
Employed	107 (26.68%)	53 (23.77%)	160 (25.64%)	0.038*
Self-employed	157 (39.15%)	100 (44.84%)	257 (41.19%)	
Student	56 (13.97%)	16 (7.17%)	72 (11.54%)	
Unemployed	81 (20.20%)	54 (24.22%)	135 (21.63%)	
**Poverty**				
No	135 (54.00%)	79 (50.64%)	214 (52.71%)	0.51
Yes	115 (46.00%)	77 (49.36%)	192 (47.29%)	

**Table 3:** Logistic regression analysis on respondents who reported being told that they had hypertension.

Have you ever been told by a doctor or other health worker that you had hypertension? Yes vs No				
Effect		Odds Ratio Estimates	95% Wald Confidence Limits	P-value
**Sex.**	**Female vs Male**	2.335	(1.470, 3.707)	0.0003
**Education Level.**	**Less than primary school or no formal schooling vs Secondary school or above**	1.109	(0.492, 2.500)	0.80
**Education Level.**	**Primary school completed vs Secondary school or above**	1.029	(0.599, 1.767)	0.92
**Marital Status.**	**Formerly married vs Never married**	1.813	(0.702, 4.678)	0.22
**Marital Status.**	**Married vs Never married**	1.175	(0.657, 2.102)	0.59
**Work Status.**	**Employed vs Unemployed**	0.743	(0.350, 1.576)	0.44
**Work Status.**	**Self-employed vs Unemployed**	0.630	(0.313, 1.268)	0.20
**Work Status.**	**Student vs Unemployed**	0.931	(0.281, 3.077)	0.91
**Age**	**Years**	1.066	(1.041, 1.092)	< .0001
**Poverty.**	**Yes vs No**	0.607	(0.372, 0.992)	0.046

Nearly one in five patients (18.3%, n = 61/333) reported being told they had elevated blood sugar or diabetes by a health worker (see Table 4). The average age among those reporting diagnosis was 49.5y (+/- 13.9y). The mean reported income was 1,851.19 USD. The majority of respondents who reported having been diagnosed with diabetes were men (60.7%, n = 37/61), and tended towards lower levels of education with more than half (63.9%, n = 39/61) reporting primary school or less. In the multivariate analysis, there was statistically significant evidence of likelihood of diagnosis being associated with advanced age (see Tables 5 and 15), and for every 1-year increase in age, the odds of having been told that they had raised blood sugar or diabetes increased by 0.072 (7.2%) (95% CI 1.032–1.113, p = 0.0003).

**Table 4:** Sociodemographic characteristics of respondents told by a doctor or other health worker that they had raised blood sugar or diabetes.

	No (N = 272)	Yes (N = 61)	Total (N = 333)	P Value
**Age**				
Mean (SD)	38.38 (13.42)	49.48 (13.89)	40.43 (14.16)	<0.001***
**Household income (Ksh)**				
Mean (SD)	265718.23 (285027.30)	185119.05 (211180.68)	250538.12 (274052.37)	0.041*
**Household size**				
Mean (SD)	2.62 (1.50)	3.44 (2.77)	2.77 (1.83)	0.027*
**Sex**				
Female	125 (45.96%)	24 (39.34%)	149 (44.74%)	0.35
Male	147 (54.04%)	37 (60.66%)	184 (55.26%)	
**Education Level**				
Less than primary school or none	31 (11.48%)	20 (32.79%)	51 (15.41%)	<0.001***
Primary school completed	75 (27.78%)	19 (31.15%)	94 (28.40%)	
Secondary school or above	164 (60.74%)	22 (36.07%)	186 (56.19%)	
**Marital Status**				
Formerly Married	30 (11.15%)	12 (19.67%)	42 (12.73%)	0.07
Married	171 (63.57%)	40 (65.57%)	211 (63.94%)	
Never married	68 (25.28%)	9 (14.75%)	77 (23.33%)	
**Work Status**				
Employed	73 (27.04%)	11 (18.33%)	84 (25.45%)	0.033*
Self-employed	125 (46.30%)	33 (55.00%)	158 (47.88%)	
Student	22 (8.15%)	0 (0.00%)	22 (6.67%)	
Unemployed	50 (18.52%)	16 (26.67%)	66 (20.00%)	
**Poverty**				
No	102 (56.67%)	17 (40.48%)	119 (53.60%)	0.06
Yes	78 (43.33%)	25 (59.52%)	103 (46.40%)	

**Table 5:** Logistic regression analysis on respondents who reported being told that they had raised blood sugar or diabetes.

Have you ever been told by a doctor or other health worker that you have raised blood sugar or diabetes? Yes vs No				
Effect		Odds Ratio Estimates	95% Wald Confidence Limits	P-value
**Sex.**	**Female vs Male**	0.593	(0.262, 1.346)	0.21
**Education Level.**	**Less than primary school or no formal schooling vs Secondary school or above**	2.672	(0.922, 7.744)	0.082
**Education Level.**	**Primary school completed vs Secondary school or above**	1.251	(0.481, 3.254)	0.56
**Marital Status.**	**Formerly married vs Never married**	0.921	(0.226, 3.756)	0.94
**Marital Status.**	**Married vs Never married**	0.783	(0.258, 2.372)	0.64
**Age.**	**Years**	1.072	(1.032, 1.113)	0.0003
**Poverty.**	**Yes vs No**	0.947	(0.404, 2.221)	0.90

38.5% (298/774) of respondents reported having ever used tobacco (smoked or smokeless), and 18.2% (n = 119/654) of respondents reported being current smokers (see Table 1). The average age of users was 38y with a range from 17y to 88y, most had completed high school, and the vast majority of users were male (see Table 6). Male sex was a strong predictor of tobacco use, with an eight times (OR 8.86, 95% CI 5.2–15.1) higher odds of use as compared to their female counterparts (see Tables 7 and 15). Additionally, lower levels of education were more predictive of tobacco use, with those having completed less than primary school being more likely to engage in tobacco use as compared to those that had completed secondary school or above (OR 6.0, 95% CI 2.808–12.618, p < 0.0001), adjusting for other covariates. 40.8% (n = 316/774) of respondents reported being exposed to smoke in home, and 47.7% reported exposure (n = 374/776) at work.

**Table 6:** Sociodemographic characteristics of respondents who reported having used tobacco (smoked or smokeless).

	No (N = 476)	Yes (N = 298)	Total (N = 774)	P Value
**Age**				
Mean (SD)	33.39 (12.14)	38.24 (13.69)	35.28 (12.97)	<0.001***
**Household income (Ksh)**				
Mean (SD)	239245.95 (278069.92)	231780.91 (249729.78)	236286.18 (266968.18)	0.76
**Household size**				
Mean (SD)	2.69 (1.58)	2.58 (2.36)	2.65 (1.92)	0.48
**Sex**				
Female	251 (52.95%)	46 (15.44%)	297 (38.47%)	<0.001***
Male	223 (47.05%)	252 (84.56%)	475 (61.53%)	
**Education Level**				
Less than primary school or none	37 (7.79%)	65 (21.89%)	102 (13.21%)	<0.001***
Primary school completed	123 (25.89%)	87 (29.29%)	210 (27.20%)	
Secondary school or above	315 (66.32%)	145 (48.82%)	460 (59.59%)	
**Marital Status**				
Formerly Married	32 (6.77%)	36 (12.12%)	68 (8.83%)	0.033*
Married	262 (55.39%)	161 (54.21%)	423 (54.94%)	
Never married	179 (37.84%)	100 (33.67%)	279 (36.23%)	
**Work Status**				
Employed	122 (25.74%)	77 (26.01%)	199 (25.84%)	<0.001***
Self-employed	197 (41.56%)	123 (41.55%)	320 (41.56%)	
Student	76 (16.03%)	19 (6.42%)	95 (12.34%)	
Unemployed	79 (16.67%)	77 (26.01%)	156 (20.26%)	
**Poverty**				
No	159 (51.62%)	113 (55.67%)	272 (53.23%)	0.37
Yes	149 (48.38%)	90 (44.33%)	239 (46.77%)	

**Table 7:** Logistic regression analysis on respondents who reported having used tobacco.

Have you ever used tobacco? Yes vs No				
Effect		Odds Ratio Estimates	95% Wald Confidence Limits	P-value
**Sex.**	**Female vs Male**	0.117	(0.069, 0.199)	< .0001
**Education Level.**	**Less than primary school or no formal schooling vs Secondary school or above**	5.952	(2.808, 12.618)	< .0001
**Education Level.**	**Primary school completed vs Secondary school or above**	1.472	(0.9, 2.407)	0.12
**Marital Status.**	**Formerly married vs Never married**	1.083	(0.435, 2.697)	0.86
**Marital Status.**	**Married vs Never married**	1.059	(0.625, 1.795)	0.83
**Work Status.**	**Employed vs Unemployed**	0.573	(0.272, 1.209)	0.14
**Work Status.**	**Self-employed vs Unemployed**	0.594	(0.295, 1.197)	0.15
**Work Status.**	**Student vs Unemployed**	0.362	(0.127, 1.028)	0.056
**Age**	**Years**	1.020	(0.999, 1.042)	0.059
**Poverty.**	**Yes vs No**	0.664	(0.423, 1.04)	0.074

Another 61.6% (477/774) reported having used alcohol, majority being current users, having consumed alcohol within the past year. The average age of alcohol users was 36y, and majority of users were also male (see Table 8). Females have 0.324 (95% CI 0.215–0.488, p < .0001) times the odds of ever consuming any alcohol, compared to males, adjusting for other covariates (see Tables 9 and 15). Those who completed primary school have 0.620 (95% CI 0.386–0.994, p = 0. 0472) times the odds, compared to those who attended secondary school or above.

**Table 8:** Sociodemographic characteristics of respondents who reported having consumed any alcohol within the past 12 months.

	No (N = 200)	Yes (N = 276)	Total (N = 476)	P Value
**Age**				
Mean (SD)	41.31 (13.78)	32.24 (10.40)	36.04 (12.74)	<0.001***
**Household income (Ksh)**				
Mean (SD)	245283.39 (257736.17)	247225.64 (262612.57)	246403.92 (260177.74)	0.95
**Household size**				
Mean (SD)	2.85 (1.81)	2.45 (2.21)	2.62 (2.06)	0.033*
**Sex**				
Female	69 (34.50%)	53 (19.27%)	122 (25.68%)	<0.001***
Male	131 (65.50%)	222 (80.73%)	353 (74.32%)	
**Education Level**				
Less than primary school or none	33 (16.58%)	32 (11.59%)	65 (13.68%)	0.11
Primary school completed	52 (26.13%)	61 (22.10%)	113 (23.79%)	
Secondary school or above	114 (57.29%)	183 (66.30%)	297 (62.53%)	
**Marital Status**				
Formerly Married	19 (9.60%)	21 (7.61%)	40 (8.44%)	<0.001***
Married	137 (69.19%)	130 (47.10%)	267 (56.33%)	
Never married	42 (21.21%)	125 (45.29%)	167 (35.23%)	
**Work Status**				
Employed	57 (28.79%)	73 (26.45%)	130 (27.43%)	0.012*
Self-employed	85 (42.93%)	117 (42.39%)	202 (42.62%)	
Student	10 (5.05%)	38 (13.77%)	48 (10.13%)	
Unemployed	46 (23.23%)	48 (17.39%)	94 (19.83%)	
**Poverty**				
No	67 (46.85%)	121 (62.05%)	188 (55.62%)	0.005**
Yes	76 (53.15%)	74 (37.95%)	150 (44.38%)	

**Table 9:** Logistic regression analysis on respondents who reported having ever consumed alcohol.

Have you ever consumed any alcohol? Yes vs No				
Effect		Odds Ratio Estimates	95% Wald Confidence Limits	P-value
**Sex.**	**Female vs Male**	0.324	(0.215, 0.488)	< .0001
**Education Level.**	**Less than primary school or no formal schooling vs Secondary school or above**	1.340	(0.658, 2.728)	0.42
**Education Level.**	**Primary school completed vs Secondary school or above**	0.620	(0.386, 0.994)	0.047
**Marital Status.**	**Formerly married vs Never married**	0.887	(0.395, 1.994)	0.77
**Marital Status.**	**Married vs Never married**	1.535	(0.933, 2.525)	0.092
**Work Status.**	**Employed vs Unemployed**	1.028	(0.527, 2.007)	0.94
**Work Status.**	**Self-employed vs Unemployed**	1.120	(0.602, 2.084)	0.72
**Work Status.**	**Student vs Unemployed**	0.885	(0.358, 2.186)	0.79
**Age**	**Years**	1.010	(0.99, 1.031)	0.33
**Poverty.**	**Yes vs No**	0.836	(0.551, 1.269)	0.40

Majority of respondents had either had their blood pressure (80.3%, n = 630/772), blood sugar (42.6%, n = 334/767) or cholesterol (13.9%, n = 109/766) measured (see Tables 10–12). Of those that had been told they had elevated blood pressure, 51.6% (n = 116/225) reported taking medications. Age was the only sociodemographic variable that had statistically significant evidence predicting likelihood of using antihypertensives (see Tables 13 and 15). For every one year increase in age, the odds of ever having taken medication for hypertension in the past two weeks increased by 0.089 (8.9%) (95% CI 1.044–1.136, p <0.0001), adjusting for other covariates. Furthermore, of those that had been told they had elevated blood sugar or diabetes, 41% (n = 25/61) reported taking insulin. Again, age was the only predictor with statistical evidence for association with taking insulin in diabetic patients, with the odds of use increasing with age (OR 1.3, 95%CI 1.1–1.6, p = 0.0115), adjusting for other covariates (see Tables 14 and 15). There were no statistically evident predictors of taking statins or aspirin. While more than one in ten respondents reported a history of cardiovascular disease–either angina, heart disease or stroke (11.7%, n = 90/769), only one in four (23.3%, n = 21/90) of these were taking aspirin and one in six (17.44%, n = 15/86) were taking a statin. Only 3 CVD patients were taking both an aspirin and statin.

**Table 10:** Sociodemographic characteristics of respondents who reported having ever had their blood pressure measured by a doctor or other health worker.

	No (N = 142)	Yes (N = 630)	Total (N = 772)	P Value
**Age**				
Mean (SD)	29.70 (8.68)	36.53 (13.43)	35.25 (12.95)	<0.001***
**Household income (Ksh)**				
Mean (SD)	195892.40 (164019.98)	248629.58 (289709.91)	238102.79 (270034.14)	0.016*
**Household size**				
Mean (SD)	2.28 (1.22)	2.73 (2.04)	2.65 (1.92)	<0.001***
**Sex**				
Female	24 (16.90%)	275 (43.79%)	299 (38.83%)	<0.001***
Male	118 (83.10%)	353 (56.21%)	471 (61.17%)	
**Education Level**				
Less than primary school or none	22 (15.49%)	79 (12.58%)	101 (13.12%)	0.55
Primary school completed	40 (28.17%)	168 (26.75%)	208 (27.01%)	
Secondary school or above	80 (56.34%)	381 (60.67%)	461 (59.87%)	
**Marital Status**				
Formerly Married	10 (7.04%)	56 (8.95%)	66 (8.59%)	0.009**
Married	65 (45.77%)	360 (57.51%)	425 (55.34%)	
Never married	67 (47.18%)	210 (33.55%)	277 (36.07%)	
**Work Status**				
Employed	39 (27.46%)	160 (25.56%)	199 (25.91%)	0.14
Self-employed	60 (42.25%)	259 (41.37%)	319 (41.54%)	
Student	23 (16.20%)	72 (11.50%)	95 (12.37%)	
Unemployed	20 (14.08%)	135 (21.57%)	155 (20.18%)	
**Poverty**				
No	57 (55.88%)	214 (52.45%)	271 (53.14%)	0.53
Yes	45 (44.12%)	194 (47.55%)	239 (46.86%)	

**Table 11:** Sociodemographic characteristics of respondents who reported having ever had their blood sugar measured by a doctor or other health worker.

	No (N = 433)	Yes (N = 334)	Total (N = 767)	P Value
**Age**				
Mean (SD)	31.19 (10.11)	40.43 (14.14)	35.18 (12.85)	<0.001***
**Household income (Ksh)**				
Mean (SD)	225626.40 (257991.74)	250538.12 (274052.37)	236562.06 (265185.17)	0.29
**Household size**				
Mean (SD)	2.55 (1.99)	2.77 (1.82)	2.64 (1.92)	0.11
**Sex**				
Female	148 (34.34%)	150 (44.91%)	298 (38.95%)	0.003**
Male	283 (65.66%)	184 (55.09%)	467 (61.05%)	
**Education Level**				
Less than primary school or none	50 (11.55%)	51 (15.36%)	101 (13.20%)	0.15
Primary school completed	113 (26.10%)	95 (28.61%)	208 (27.19%)	
Secondary school or above	270 (62.36%)	186 (56.02%)	456 (59.61%)	
**Marital Status**				
Formerly Married	24 (5.56%)	42 (12.69%)	66 (8.65%)	<0.001***
Married	210 (48.61%)	212 (64.05%)	422 (55.31%)	
Never married	198 (45.83%)	77 (23.26%)	275 (36.04%)	
**Work Status**				
Employed	114 (26.33%)	84 (25.45%)	198 (25.95%)	<0.001***
Self-employed	159 (36.72%)	158 (47.88%)	317 (41.55%)	
Student	72 (16.63%)	22 (6.67%)	94 (12.32%)	
Unemployed	88 (20.32%)	66 (20.00%)	154 (20.18%)	
**Poverty**				
No	151 (52.98%)	119 (53.60%)	270 (53.25%)	0.89
Yes	134 (47.02%)	103 (46.40%)	237 (46.75%)	

**Table 12:** Have you ever had your cholesterol (fat levels in your blood) measured by a doctor or other health worker?.

	No (N = 657)	Yes (N = 109)	Total (N = 766)	P Value
**Age**				
Mean (SD)	33.95 (12.06)	42.74 (15.33)	35.16 (12.91)	<0.001***
**Household income (Ksh)**				
Mean (SD)	224326.85 (248126.70)	324800.00 (364173.96)	239160.48 (270350.41)	0.024*
**Household size**				
Mean (SD)	2.57 (1.62)	3.17 (3.19)	2.65 (1.93)	0.06
**Sex**				
Female	247 (37.71%)	51 (46.79%)	298 (39.01%)	0.07
Male	408 (62.29%)	58 (53.21%)	466 (60.99%)	
**Education Level**				
Less than primary school or none	88 (13.41%)	13 (12.04%)	101 (13.22%)	0.53
Primary school completed	180 (27.44%)	25 (23.15%)	205 (26.83%)	
Secondary school or above	388 (59.15%)	70 (64.81%)	458 (59.95%)	
**Marital Status**				
Formerly Married	51 (7.80%)	14 (12.96%)	65 (8.53%)	0.001**
Married	350 (53.52%)	71 (65.74%)	421 (55.25%)	
Never married	253 (38.69%)	23 (21.30%)	276 (36.22%)	
**Work Status**				
Employed	163 (24.92%)	33 (30.56%)	196 (25.72%)	0.37
Self-employed	270 (41.28%)	46 (42.59%)	316 (41.47%)	
Student	86 (13.15%)	9 (8.33%)	95 (12.47%)	
Unemployed	135 (20.64%)	20 (18.52%)	155 (20.34%)	
**Poverty**				
No	226 (52.31%)	45 (60.00%)	271 (53.45%)	0.22
Yes	206 (47.69%)	30 (40.00%)	236 (46.55%)	

**Table 13:** Logistic regression analysis on respondents who reported having taken medication for hypertension.

In the past two weeks have you taken medication for hypertension? Yes vs No				
Effect		Odds Ratio Estimates	95% Wald Confidence Limits	P-value
**Sex.**	**Female vs Male**	0.524	(0.233, 1.181)	0.12
**Education Level.**	**Less than primary school or no formal schooling vs Secondary school or above**	1.314	(0.381, 4.538)	0.67
**Education Level.**	**Primary school completed vs Secondary school or above**	0.889	(0.353, 2.241)	0.80
**Marital Status.**	**Formerly married vs Never married**	0.482	(0.108, 2.145)	0.34
**Marital Status.**	**Married vs Never married**	0.621	(0.222, 1.734)	0.36
**Work Status.**	**Employed vs Unemployed**	0.565	(0.158, 2.027)	0.38
**Work Status.**	**Self-employed vs Unemployed**	0.854	(0.272, 2.680)	0.79
**Work Status.**	**Student vs Unemployed**	0.332	(0.024, 4.523)	0.41
**Age.**	**Years**	1.089	(1.044, 1.136)	< .0001
**Poverty.**	**Yes vs No**	1.254	(0.515, 3.051)	0.62

**Table 14:** Logistic regression analysis on respondents who reported taking insulin.

Are you currently taking insulin? Yes vs No				
Effect		Odds Ratio Estimates	95% Confidence Limits	p-Value
**Sex.**	**Female vs Male**	0.806	(0.095, 6.862)	0.84
**Education Level.**	**Less than primary school or no formal schooling vs Secondary school or above**	0.063	(0.001, 2.779)	0.15
**Education Level.**	**Primary school completed vs Secondary school or above**	1.433	(0.07, 29.185)	0.81
**Marital Status.**	**Formerly married vs Never married**	0.277	(0.007, 11.35)	0.50
**Marital Status.**	**Married vs Never married**	0.061	(0.001, 2.541)	0.14
**Age.**	**Years**	1.32	(1.064, 1.637)	0.012
**Poverty.**	**Yes vs No**	4.187	(0.274, 63.935)	0.30

**Table 15:** Sociodemographic factors that had statistically significant relationships with outcomes in regression analyses (outcomes: Presence of hypertension, raised blood sugar or diabetes, tobacco use, alcohol use, taking medications).

**Effect**		**Odds Ratio Estimates**	**95% Wald Confidence Limits**	**P-value**
**Have you ever been told by a doctor or other health worker that you had hypertension? Yes vs No**				
**Sex.**	**Female vs Male**	2.335	(1.470, 3.707)	0.0003
**Age.**	**Years**	1.066	(1.041, 1.092)	< .0001
**Poverty.**	**Yes vs No**	0.607	(0.372, 0.992)	0.046
**Have you ever been told by a doctor or other health worker that you have raised blood sugar or diabetes? Yes vs No**				
**Education Level.**	**Less than primary school or no formal schooling vs Secondary school or above**	2.672	(0.922, 7.744)	0.082
**Age.**	**Years**	1.072	(1.032, 1.113)	0.0003
**Have you ever used tobacco? Yes vs No**				
**Sex.**	**Female vs Male**	0.117	(0.069, 0.199)	< .0001
**Education Level.**	**Less than primary school or no formal schooling vs Secondary school or above**	5.952	(2.808, 12.618)	< .0001
**Have you ever consumed any alcohol? Yes vs No**				
**Sex.**	**Female vs Male**	0.324	(0.215, 0.488)	< .0001
**Education Level.**	**Primary school completed vs Secondary school or above**	0.620	(0.386, 0.994)	0.047
**In the past two weeks have you taken medication for hypertension? Yes vs No**				
**Age.**	**Years**	1.089	(1.044, 1.136)	< .0001
**Are you currently taking insulin? Yes vs No**				
**Age.**	**Years**	1.32	(1.064, 1.637)	0.012

## Discussion

In this study, we described the burden of NCDs and mental health at the Kenyatta National Hospital Emergency Department, the largest hospital in East Africa. More than a third of respondents had hypertension, one in five had raised blood sugar or diabetes, and more than one in ten reported having cardiovascular disease. More than one third reported tobacco use, and two thirds reported alcohol use. Majority reported not taking medications despite diagnosis, with the highest proportion being half of those diagnosed with hypertension reporting taking medication. Determinants of disease burden were age, sex, and income. Having lower levels of education was associated with tobacco use, however those with higher levels of education reported alcohol use. While a predominant proportion of respondents had had some form of screening for either hypertension, diabetes, or high cholesterol, the proportion of those on treatment was low. Our results showed a systematically higher NCD burden among the ED population as compared to the national population as demonstrated by the Kenya national Stepwise approach to Surveillance (STEPs) study conducted 2 years prior [11] where the prevalence of hypertension was only in a quarter of the population, less than 3% had raised blood sugar or diabetes, only 13% reported tobacco use and 19% reported alcohol use. The mental health results from our study will be presented elsewhere.

There was a higher prevalence of hypertension in our study on an ED population (35.8%) as compared to the national STEPs study (24.5%) utilizing the same validated survey tool [13], and as compared to the prevalence of national populations in the surrounding region: 24.3% in Uganda [14], 26% in Tanzania [15], and 11.2% in Rwanda [16]. The same is true for diabetes with only 2.1% of the general population reporting a diabetes diagnosis nationally in the Kenya STEPs study [11]. This high prevalence of disease in the ED population is well-established in the US, Canada and UK settings [17]. While the population presenting to the ED are likely to have higher prevalence of disease as this is a facility-based setting, and results may be biased by patients seeking care with known disease, this still establishes the ED as a high-risk population with potential for high-impact if targeted interventions are implemented, particularly in populations that may not otherwise access the healthcare system. A systematic review and meta-analysis by Armitage et al demonstrates the opportunity for detection of hypertension during screening in the ED [17]. This in turn provides the opportunity for patient education, linkage to care, or prescription of medication through the ED, and could affect immediate care such as screening for end-organ damage during the acute visit [4].

The average age of those diagnosed with hypertension was 42y. This is in contrast to US-based populations where hypertension is predominantly prevalent in those 60 years and older, as demonstrated by data from the National Health and Nutrition Examination Survey (NHANES) [18]. However, this observation of afflicted younger populations is not uncommon in African populations. Results from the Rwanda STEPS demonstrated a hypertension prevalence of 13% among 25–34 year olds, and 19% among 35–44 year olds [16]. The case is comparable for diabetes, with average age of diagnosis in our sample being 49y. Similarly, in the Rwanda case, the distribution of diabetes did not show significant difference for younger individuals. Our data support the need for greater attention to younger adults in this setting, as opposed to recommendations traditionally targeting only those 40 years and older [19]. By implementing primary and secondary prevention efforts for these younger groups the costly implications of NCD complications can be mitigated.

Women had a higher likelihood of hypertension diagnosis. The odds of women reporting a higher prevalence of disease is divergent from most African countries where there is a male predominance [20]. In the national STEPS study, men had much higher likelihood of not having had their blood pressure taken (71.1%, 95% CI 64.9–77.3) as compared to women (41.3%, 95% CI 36.5–46.0). Just as in our ED sample population, there was also a higher proportion of women that reported having been diagnosed as compared to men. Contrary to this, there was a predominance of men who reported having been diagnosed with diabetes (60.7%) in our study, whereas in the national data, prevalence of disease was equal among both sexes. These findings highlight an interesting and important disparity in care-seeking behaviors of men, which may not occur unless symptoms exist. This is particularly detrimental for hypertension that tends to present asymptomatically [21]. To that end, increased attention is needed for screening and diagnosis of men during clinical encounters, including in the safety net of the ED while continuing to advance community-based prevention efforts.

Those below the poverty line had 40% less likelihood of being diagnosed with hypertension. Additionally, among all patients that reported having a diagnosis of hypertension, diabetes, high cholesterol or CVD, majority had a secondary school education or higher. These markers of socioeconomic status raise concerns about diagnosis and management of disease among marginalized populations in the ED. The *Lancet* Commission on Reframing NCDs and Injuries (NCDI) for the Poorest Billion highlights poverty as a primary driver for NCDs [22]. In our study, the average reported annual household income was Kenya Shillings (Kshs) 237,888.6, or approximately 2,379 USD, with 30.6% falling below the World Bank international poverty line of 1.90 USD per day (n = 240/784). The Lancet Commission has facilitated development of “national NCDI poverty commissions”, as well as leveraging the WHO PEN-Plus package–two interventions occurring at the policy level that could help facilitate sustainable change for individuals affected by NCDs in LMICs [23].

One in five Kenyans reported having used tobacco (21.3%) in the national STEPS study [11], whereas 38.5% reported use in our study. Nearly half of individuals reported exposure to smoke in the workplace or at home. In sum, exposure to tobacco is a significant problem in this population, and an alarming burden of disease is attributable to tobacco alone [24]. The WHO Tobacco Free Initiative highlights the role for brief tobacco interventions in healthcare settings, which have demonstrated effectiveness in US-based ED populations [5], but there are no studies to date on interventions in an African ED.

Overall, the prevalence of alcohol use was remarkably high. Those with higher levels of education were more likely to report engaging in alcohol use. This observation is divergent from tobacco use, which is more likely among those with lower levels of education. This phenomenon of education being associated with increased alcohol use has been demonstrated in other settings, including the US, however the downstream effects of alcohol use such as morbidity and mortality of disease remains disproportionately higher in those of lower socioeconomic status [25], and public health programming should be targeted accordingly. Brief interventions on alcohol intake in clinical settings are another intervention with demonstrated success in the ED setting [3].

Among those diagnosed with hypertension or diabetes, two of the leading NCDs, the vast majority were not taking medications for disease. In our study, the only determinant of using medications for NCDs and NCD risk factors was age. However, the lack of statistical evidence of differences in medication use compared across socioeconomic determinants likely indicates a universal lack of access regardless of sex, education and poverty level. Alternatively, it is possible that differences were not detected in some cases due to relatively small sample sizes for these questions. Our findings highlight the need to develop interventions to increase appropriate use of medication for individuals younger than 50y. Prescription of antihypertensives at ED discharge has been shown to improve blood pressure with no significant detrimental side effects in follow-up [26]. Mobilizing the ED to initiate, and help ensure compliance with, medications should be prioritized.

### Next steps

Clinical protocols should be developed and routinely reviewed with providers, to help standardize therapy and empower education on self-care practices for patients. Education on hypertension and diabetes can also be facilitated by staff, and task-shifting can be employed such as through use of navigators—dedicated, trained nurses or lay health workers. Additionally, linkage to care programs such as those connecting to Community Health Workers could be implemented [27]. Mobile health (mHealth) technology presents a unique low-cost, highly effective opportunity to enhance health education, support self-monitoring, and improve follow-up [28], which could also be leveraged in ED populations. National policies that further address availability and affordability of medications are needed, in addition to ensuring enforcement of the WHO essential medicines list with first-line blood pressure and diabetes medications. In addition, efforts beyond the ED include media campaigns, and strengthening primary healthcare. WHO initiatives such as the SAFER technical package address alcohol access, and include strengthening restrictions on alcohol availability, enforcing restrictions on alcohol advertising and promotion, among other recommendations [29]. Finally, our findings highlight the importance of legislation that implements and enforces smoke-free zones in public places and workplaces, as well as regulates how these policies are enforced with clear sanctions for entities not abiding by guidelines [30].

### Limitations

The sample studied in our population may not have findings generalizable to the national population given the study was conducted at a tertiary, referral hospital which may represent a population with higher prevalence of comorbidities. Also, given that this is a pilot study using a convenience sample, a nationally randomized control study would be beneficial to provide greater generalizability of results. Additionally, certain sub-analyses had too few respondents and so may have affected our ability to detect differences in this population, including the relative minority of women and our ability to detect further potential sex differences. With that said, in selecting a pilot study site, we felt that this site was likely to provide one of the most optimal sites to capture patients presenting from across the region, and our results provide novel evidence of concerning disease prevalence in a large population that seeks care here. Furthermore, our study provides insight into some of the most economically challenged populations in Kenya who receive care in the public health system, which is where the majority commonly seek care in Kenya and Africa at large. Finally, our findings provide incipient insights into a problem that has otherwise gone unaddressed, which will ideally generate opportunities for future research.

## Conclusions

The ED acts as a catchment site for patients that may not otherwise frequent a healthcare setting. Comprehension of the unique epidemiology and characteristics of patients presenting to the ED is key in order to guide care. Opportunities exist for further research including longitudinal data collection through surveillance and registries to better understand epidemiology of disease, as well as implementation science to guide effective intervention planning. Patient-driven interventions, and collaboration with community-based stakeholders such as CHWs, would be ideal considerations to sustainably address NCDs leveraging the ED in the resource-limited setting.

## Supporting information

S1 File STROBE checklist for cross-sectional studies. The STROBE checklist includes a list of items that should be included in reports of cross-sectional studies and is referred to in the Uniform Requirements for Manuscripts Submitted to Biomedical Journals by the International Committee of Medical Journal Editors.
(DOC)
